# Understanding Machining Process Parameters and Optimization of High-Speed Turning of NiTi SMA Using Response Surface Method (RSM) and Genetic Algorithm (GA)

**DOI:** 10.3390/ma16175786

**Published:** 2023-08-24

**Authors:** Yanzhe Zhao, Li Cui, Vinothkumar Sivalingam, Jie Sun

**Affiliations:** 1 School of Intelligent Manufacturing and Control Engineering, Shanghai Polytechnic University, Shanghai 201209, China; yzzhao@sspu.edu.cn; 2 Key Laboratory of High-Efficiency and Clean Mechanical Manufacture, National Demonstration Center for Experimental Mechanical Engineering Education, School of Mechanical Engineering, Shandong University, Jinan 250061, China; sunjie@sdu.edu.cn; 3 Department of Mechanical Engineering, Saveetha School of Engineering, SIMATS, Chennai 602105, India

**Keywords:** RSM, surface roughness, remnant depth ratio, multi-objective optimization, genetic algorithm

## Abstract

This study aimed to optimize machining parameters to obtain better surface roughness and remnant depth ratio values under dry turning of NiTi-shape memory alloy (SMA). During the turning experiments, various machining parameters were used, including three different cutting speeds *v_c_* (105, 144, and 200 m/min), three different feed rates *f* (0.05, 0.1, and 0.15 mm/rev), and three different depths of cut *a_p_* (0.1, 0.15, and 0.2 mm). The effects of machining parameters in turning experiments were investigated on the response surface methodology (RSM) with Box–Behnken design (BBD) using the Design Expert 11; how the cutting parameters affect the surface quality is discussed in detail. In this context, the cutting parameters were successfully optimized using a genetic algorithm (GA). The optimized processing parameters are *v_c_* = 126 m/min, *f* = 0.11 mm/rev, *a_p_* = 0.14 mm, resulting in surface roughness and remnant depth ratio values of 0.489 μm and 64.13%, respectively.

## 1. Introduction

Nickel-Titanium (NiTi) shape memory alloy (SMA) is widely used in aerospace, automotive, medical, and other fields because of its excellent performance [1]. The shape memory effect and superelasticity are popular features of NiTi SMA [2]. Certain types of shape memory alloys exhibit unique behaviors that result from mechanical or thermal actions [3]. The clamping devices, medical guidewire, and aerospace are made based on their properties [4,5,6,7,8]. The higher shape adaptability of these products needs a fine surface finish, which raises the requirements for machining NiTi SMA. As a common way in traditional processing, turning is also an important processing means of NiTi SMA. Efficient and green machining of NiTi SMA has always been the goal of researchers.

The transformation between the martensite and austenite phases results in its shape memory effect and superelasticity [9]. During machining, this material experiences a solid-state phase transformation. However, the different characteristics of martensite and austenite phases also bring difficulties to machining NiTi SMA, such as tool wear, poor surface quality, work hardening, etc. [10,11,12]. Different optimization methods have been applied to improve the machinability of NiTi SMA. Weinert et al. [13] applied different cutting tools to study the turning and drilling processes of different Nitinol materials. Through the analysis of tool wear, the optimal parameters are put forward, and a process for turning NiTiNb pipe joints on this basis was proposed. Biermann et al. [14] proposed a simulation algorithm to optimize tool inclination angle in the micro-milling process based on the geometric analysis of meshing conditions of cutting edges. And an optimization strategy was also proposed in the drilling process: the cutting speed (*v_c_*) should be less than 30 m/min, and the feed is 5 μm when the tool diameter is 1 mm. Kuppuswamy et al. [15] designed the Taguchi experiment to provide optimal cutting conditions for Nitinol micro-milling to minimize cutting force and burr size. The order of influence on reducing the cutting force and burr size is cutting speed (*v_c_*), feed (*f*), and depth of cut (*a_p_*). And when the cutting speed is 15 m/min, the cutting force is low, and the burr size decreases. Kowalczyk [16] used the Taguchi method to turn precision and micro-mill NiTi SMA. The Signal-to-Noise Ratio (S/N), ANOVA, and Monte Carlo method were used to optimize the cutting parameters to minimize surface roughness (*R_a_*) [17], cutting temperature (CT) [18], and cutting force (CF) [19], respectively. Moreover, the Monte Carlo method was also used to turn NiTi alloy to establish the surface roughness model [20]. Wang et al. [21] designed the orthogonal experiment for milling Ni50.8Ti SMA and obtained the optimal cutting parameters for the minimum work hardening. And the influence of cutting parameters on surface roughness, microhardness, and strain hardening was analyzed by statistics [22]. The cutting speed has the greatest influence on surface roughness and strain hardening. Kabil et al. [23] used genetic algorithm (GA) to optimize the cutting parameters for NiTiHf high-temperature SMA to obtain maximum tool life, minimum energy consumption, and maximum surface quality. Kaynak et al. [24] used three different conditions to compare the tool wear and surface quality for turning NiTiHf high-temperature SMA; the condition greatly affects the machinability.

Determining the optimized cutting parameters is important in enhancing production efficiency and the overall service performance of products. Previous studies have primarily concentrated on optimizing machining parameters related to cutting force, cutting temperature, surface roughness, and work hardening. The optimized parameters are suggested, but a detailed assessment of superelasticity and shape memory effect is rarely mentioned. Because the unique properties of SMA are the main reason they are widely used, the superelasticity and shape memory effect should be checked after machining. And the level of their indicators is also one of the important factors in the quality of processing.

Upon analyzing the relevant literature on the prediction and optimization of NiTi SMA machining processes, it is evident that the current trend leans towards the utilization of orthogonal experiment, GA, and Monte Carlo method. The response surface methodology (RSM) establishes regression equations in fewer experiments and accurately analyzes and identifies key factors affecting process or product performance. And visualized results help engineers and decision makers better understand the process or product performance characteristics and make effective decisions.

Considering the above literature comprehensively, this paper presents a multi-objective optimization method to obtain minimizing surface roughness and maximizing superelasticity. The superelasticity can be measured by the remnant depth ratio ($\eta_{p}$) [25], so the regression equations of surface roughness and remnant depth ratio are established by the turning experiment of NiTi SMA based on RSM. The optimized parameters are cutting speed (126 m/min), feed rate (0.11 mm/rev), and depth of cut (0.14 mm). The influence of machining parameters on superelasticity is analyzed for the first time. The optimized parameters based on GA are not only for the surface roughness, but also for the superelasticity, which has never been studied. This study provides valuable insights into the optimization of the turning process for NiTi SMA, which can enhance the accuracy and efficiency of the manufacturing process and improve the quality of SMA products.

## 2. Experimental Section

### 2.1. Materials

The material was a Ni50.8Ti SMA solid cylindrical bar with a size of Φ80 × 8 mm. In order to unify the variables, a 2.5-mm-wide feed slot was cut into the pipe for the engage motion first, and the cutting length of each test was 15 mm. The Netzsch DSC3500 Sirius, Selb, Germany. was used to obtain the phase transition temperature. The austenite finish temperature (*A_f_*) is −2 °C, so it is the austenitic phase at room temperature. The emissivity of the sample was 0.21 [26].

**Figure 1:**
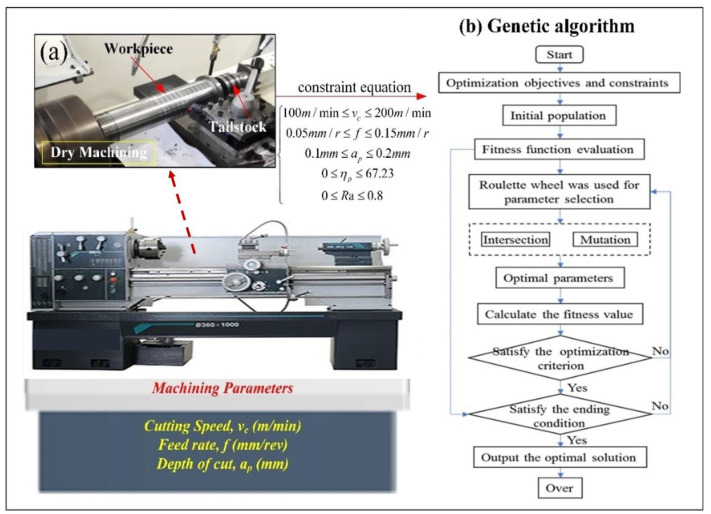
The schematic of the experiment (**a**) experimental setup and (**b**) flow chart of genetic algorithm.

The experiment was carried out on a CDS6132 lathe, Dalian, China and the spindle speed is 1600 r/min. The Ni50.8Ti SMA solid cylindrical bar is fixed to the lathe by the tailstock. Based on previous experiments [26,27,28], a VNMG160408-SM1105 PVD insert with TiAlN coating was used for turning NiTi SMA. The cutting speed (*v_c_*), feed rate (*f*), and depth of cut (*a_p_*) are input variables. After the prediction models of *R_a_* and $\eta_{p}$ are established, the optimal processing parameters were determined by GA, as shown in Figure 1b. A new insert was used for each test to avoid the effect of tool wear. The schematic of the experimental setup is shown in Figure 1a.

### 2.2. Measurements

**Figure 2:**
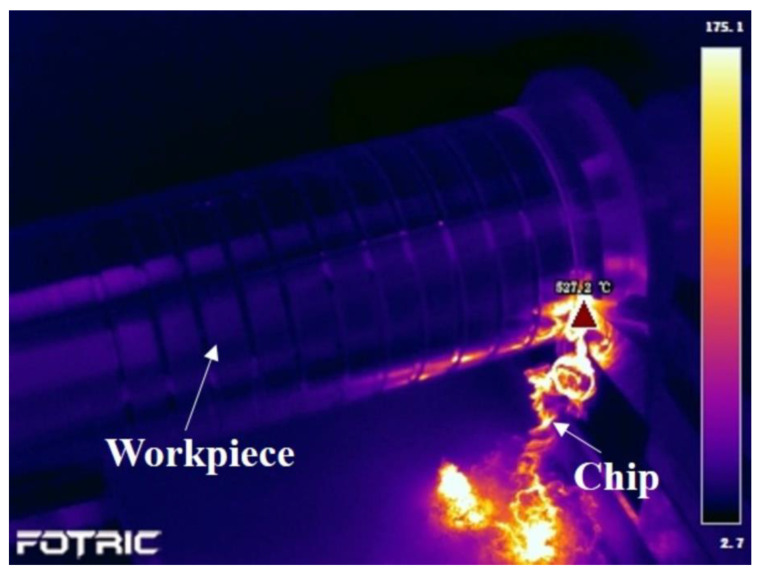
The cutting temperature when the cutting speed is 144 m/min, feed is 0.05 mm/rev, and depth of cut is 0.1 mm.

The cutting temperature is measured by FORTIC 226, and the cutting temperature of the group 1 test during machining is shown in Figure 2. The cutting temperature is between 500 °C and 1000 °C in all the tests. It is much higher than the martensitic transition temperature [29]. There is no martensitic transformation occurring during machining. The surface roughness was examined by the TR200 mobile surface roughness meter, which is produced by Beijing Cap High Technology Co., Ltd. (Beijing, China). Three measurements obtained the average surface roughness (*R_a_*).

**Figure 3:**
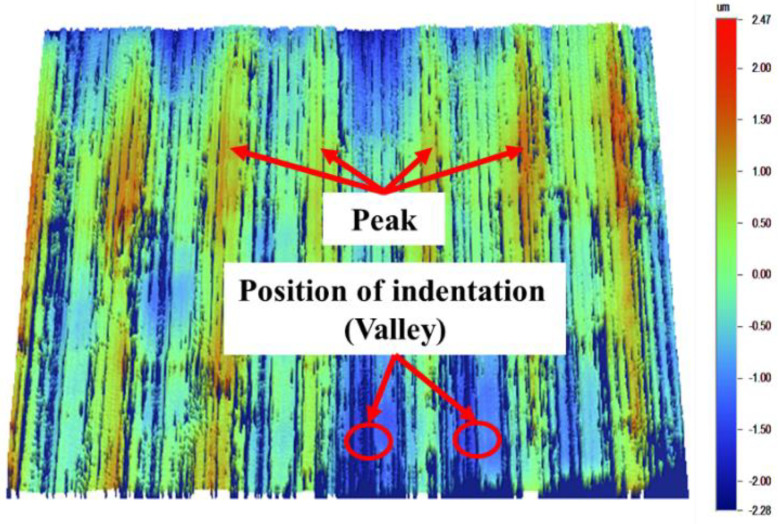
Schematic diagram of the position of indentation.

The remnant depth ratio quantified the superelasticity of NiTi SMA [25]. The nano-indentation mechanics test system can measure it. The HYSITRON TI980 (Bruker, Billerica, MA, USA) nano-indentation mechanics test system measures the force-displacement curve. A sample of 5 × 5 × 5 mm^3^ was cut from the samples by electrical discharge machining (EDM) for the nano-indentation test. Because the indenter of the nano-indentation test is very small, the indenter may press the peak or valley of surface, which will make a difference in the results of the nano-indentation. Only the lower surface position of each group of samples was selected, as shown in Figure 3. The remnant depth ratio of the as-received one was 67.23%, which is higher than that of other metals because of its superelasticity.

### 2.3. Design with Response Surface Methodology

In order to obtain more information from the smaller number of experiments, the response surface method based on Box–Behnken design (BBD) was adopted to design the machining parameters optimization experiment. For the purpose of avoiding the martensitic phase that occurred during turning [28], the higher cutting speed and lower depth of cut and feed are adopted. Each group of factors takes three levels, as shown in Table 1. There are 15 groups of experiments.

Based on polynomial regression analysis and the relationship between input variables and output response, the mathematical relation defined by RSM can be calculated by the following:

(1) $$y\left(x\right)=b_{0}+\sum_{i=1}^{p}b_{i}x_{i}+\sum_{i=1}^{p}b_{ii}x_{i}^{2}+\sum_{i=1}^{p}\sum_{j=1,i<j}^{p}b_{ij}x_{i}x_{j}$$

where $y\left(x\right)$ is the output variable, x is the input variables. $b_{0}$ is the fixed term. $b_{i}$, $b_{ii}$, and $b_{ij}$ are the coefficient of linear, quadratic, and cross-product terms, respectively.

The cutting speed, feed rate, and depth of cut are input variables. Since surface roughness is an important factor of surface quality and the remnant depth ratio can represent superelasticity, they are the output variables.

## 3. Results and Discussion

### 3.1. Prediction Model

The roughness and remnant depth ratio of the machined surface for each group are shown in Table 2. The surface roughness values are between 0.323 μm and 0.736 μm. The lower surface roughness can be obtained at high-speed cutting compared to low-speed cutting. On one hand, the low cutting speed is easy to produce a built-up edge (BUE), which increases friction and instability [30]. On the other hand, there is no martensitic phase transition occurring during the cutting process, and due to the high ductility of the martensite phase, the austenite phase is easier to cut [31]. The remnant depth ratio values range from 53.87% to 64.18%. The remnant depth ratio of the machined surface is less than the as-received one. This is because the material undergoes large plastic deformation and produces more grain defects, which reduces the superelastic properties of the material and makes the austenite phase tend to be mechanically stabilized [32,33].

Based on the results and Equation (2), the prediction models of the second-order regression equation with the *R_a_* and $\eta_{p}$ as the response variables are:

(2) $$\begin{array}{l} Ra=0.360+0.00508v_{c}-0.84f-4.32a_{p}-0.000003v_{c}^{2}+11.90f^{2}+21.50a_{p}{}^{2}\\ +0.0007v_{c}f-0.02630v_{c}a_{p}+8fa_{p} \end{array}$$

(3) $$\begin{array}{l} \eta_{p}=30.21-0.0621v_{c}+194.2f+321.7a_{p}+0.000199v_{c}^{2}-1101f^{2}-603a_{p}{}^{2}\\ +0.618v_{c}f-0.503v_{c}a_{p}-305fa_{p} \end{array}$$

Equation (3) is the prediction model of *R_a_*. The S of the prediction model is 0.02088, and it has a better prediction response effect from ANOVA and the calculation of the coded coefficients in Table 3. When the *p*-value of the factor within the 95% confidence level is less than 0.05, the effect of this factor on the response is more significant [34,35]. The significant model terms are B, C, B2, C2, and AC, in which the *p*-value is less than 0.05. From the coefficient, the feed has the greatest effect on the surface roughness, followed by the depth of cut and cutting speed. The influence of processing parameters on *R_a_* in the quadratic effect is as follows: *a_p_*^2^ > *f*^2^ > *v_c_*^2^. Among the processing parameters with interaction effects, *v_c_* and *a_p_* have the largest effect on surface roughness, followed by *f* and *a_p_*, and *v_c_* and *f* have a small effect on surface roughness, which can be ignored.

Equation (4) is the prediction model of $\eta_{p}$, and the ANOVA and the calculation of the coded coefficients in Table 4. As can be seen from the table, except for the quadratic term of cutting speed, other factors have a significant impact on the remnant depth ratio. In general, all factors have an impact on the dependent variable. The depth of cut has the greatest effect on the remnant depth ratio, followed by cutting speed and feed. In the quadratic effect, *f*^2^ has the greatest influence on $\eta_{p}$, and *v_c_*^2^ has the least influence. The influence of relevant processing parameters in interaction effect on the remnant depth ratio from large to small is as follows: *v_c_ f* > *v_c_a_p_* > *fa_p_*.

### 3.2. Effect of Machining Parameters on Surface Roughness

**Figure 4:**
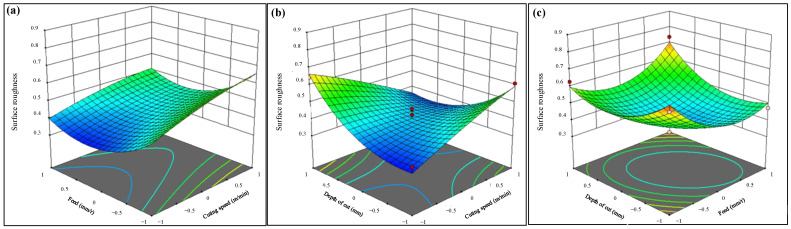
The response surface plot of surface roughness with respect to the cutting speed, feed, and depth of cut. (**a**) Effect of *v_c_* and *f* on *R_a_* (**b**) Effect of *v*_c_ and *a_p_* on *R_a_* (**c**) Effect of *f* and *a_p_* on *R_a_.*

Figure 4 shows the response surface plot of surface roughness with respect to the cutting parameters. It can be seen from Figure 4a that *v_c_* and *f* have little influence on surface roughness. At the same time, when the cutting speed is constant, the surface roughness increases significantly with the increase in the feed. The results are the same with the one at a lower cutting speed [36]. And the influence of feed on the surface roughness is greater at a higher cutting speed.

When the feed is constant, the surface roughness increases slowly with the increased cutting speed. At high temperatures, the chipping or tribo-chemical wear at the tool-workpiece interface increases the flank wear and increases the roughness [37].The effect of cutting speed on surface roughness is not different with different feeds. The interaction effect of *v_c_* and *a_p_* greatly influences the surface roughness, as shown in Figure 4b. When the cutting speed is 100 m/min, the surface roughness decreases slightly and then increases with the increase in depth of cut. With the increase in cutting speed, the surface roughness gradually decreases with the increased depth of cut. When the depth of cut is 0.1 mm, the surface roughness increases with the increased cutting speed. When the depth of cut is 0.2 mm, the surface roughness decreases gradually with the increased cutting speed. Figure 4c shows the effect of *f* and *a_p_* on surface roughness. When the feed is constant, the surface roughness decreases first and then increases with the increased depth of cut. And the surface roughness is greatly affected by the depth of cut when the feed is low. When the depth of cut is constant, the surface roughness increases with the increased feed, and the variation trend of surface roughness is the same at different depths of cut. Generally, the feed has the greatest effect on the surface roughness within the range of this experiment. A larger cutting speed, smaller feed, and depth of cut can obtain a lower surface roughness.

**Figure 5:**
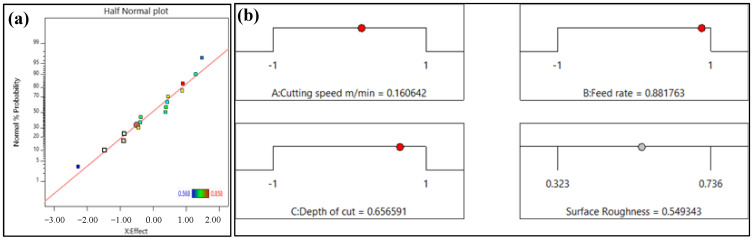
(**a**) Normal % probability and (**b**) optimum conditions for surface roughness.

The half-normal probability plot presented in Figure 5a indicates that all effect factors and interactions demonstrate significant absolute values in relation to the given grade [38,39]. This finding suggests that the selected variables have a noteworthy impact on the results observed and warrants further investigation to better understand their influence. Schematic representations of optimum conditions are given in Figure 5b. For achieving the best surface roughness of 0.5493 μm, the recommended cutting speed is 167 mm/rev, feed rate is 0.132 mm/rev, and depth of cut is 0.13 mm.

### 3.3. Effect of Machining Parameters on Remnant Depth Ratio

**Figure 6:**
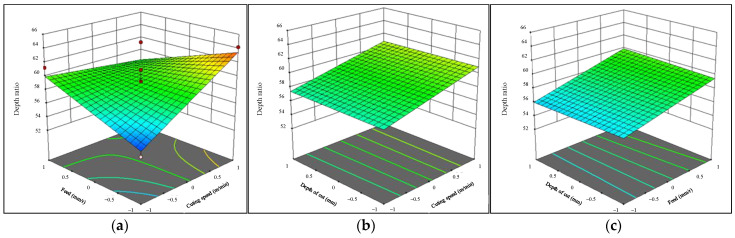
The response surface plot of remnant depth ratio with respect to the cutting speed, feed, and depth of cut. (**a**) Effect of *v_c_* and *f* on $\eta_{p}$, (**b**) Effect of *v_c_* and *a_p_* on $\eta_{p}$, (**c**) Effect of *f* and *a_p_* on $\eta_{p}$.

Figure 6 shows the response surface plot of the machining parameters to the remnant depth ratio. Figure 6a presents the remnant depth ratio with respect to cutting speed and feed. When the cutting speed is constant, the remnant depth ratio increases rapidly to the highest point with the increased feed and then decreases slowly. And the influence is greater when the cutting speed is high. When the feed is constant, the remnant depth ratio decreases with the increased cutting speed. The results are the same with that in the lower cutting speed [28]. Because of elastic energy relaxation and heterogeneous microstructure formation, the severe deformed microstructure at high speed reduced the superelasticity [36].

The decreasing trend is larger when the feed is small. It can be seen from Figure 6b that when the cutting speed is lower, the remnant depth ratio increases with the increased depth of cut. When the cutting speed is higher, the remnant depth ratio decreases with the increased depth of cut. When the depth of cut is small, the remnant depth ratio increases slowly with the increase in cutting speed. When the depth of cut is big, the remnant depth ratio decreases with the increased cutting speed. Figure 6c presents the response surface plot of the remnant depth ratio with respect to the feed and depth of cut. When the feed is 0.05 mm, the remnant depth ratio gradually increases with the increased depth of cut. With the feed increase, the remnant depth ratio first increased and then decreased with the increased depth of cut. When the depth of cut is constant, the remnant depth ratio increases first and then decreases with the increase in the feed. In total, the linear effect and the interaction effect have a greater impact on the remnant depth ratio.

**Figure 7:**
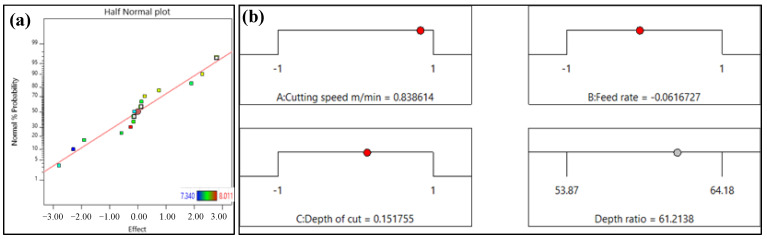
(**a**) Normal % probability and (**b**) optimum conditions for remnant depth ratio.

The half-normal probability plot shown in Figure 7a indicates that all effect factors and interactions exhibit significant absolute values with respect to the given grade. This result suggests that the chosen variables have a substantial impact on the observed outcomes, thus necessitating further investigation to gain a deeper understanding of their influence. Schematic representations of optimum conditions are given in Figure 7b. To achieve a remnant depth ratio of 61.21, it is recommended to use a cutting speed of 167 mm/rev, a feed rate of 0.106 mm/rev, and a depth of cut of 0.1727 mm.

### 3.4. Multi-Objective Optimization Using Genetic Algorithm

The smaller the surface roughness, the better the surface quality. And the bigger the remnant depth ratio, the better the performance of the superelasticity. Taking the minimum surface roughness and the maximum remnant depth ratio as the objectives, multi-objective optimization is carried out for the machining of Ni50.8Ti SMA. The optimal processing parameters are obtained based on the genetic algorithm. The optimization objective function based on the evaluation function method can be represented as the following:

(4) $$M\left(v_{c},f,a_{p}\right)=\sum_{i=1}^{2}\lambda_{i}M_{i}\left(v_{c},f,a_{p}\right)=\lambda_{R}F_{1}+\lambda_{\eta p}F_{2}\begin{array}{cc} & \left(i=1,2\right) \end{array}$$

where $\lambda_{R}$ and $\lambda_{\eta p}$ are the weighting coefficients of the surface roughness and the remnant depth ratio in the objective function, respectively. Since the surface roughness values in this parameter range are between 0.323 μm and 0.736 μm, the surface roughness has less effect on the objective function compared with the remnant depth ratio. $\lambda_{R}$ is taken to be 0.3 and $\lambda_{\eta p}$ to be 0.7.

The flow chart of the genetic algorithm is shown in Figure 1b. After the optimized objectives and constraints are determined, the parameters of the genetic algorithm are set to generate the initial population. And then the fitness of individuals in the population is calculated to judge whether the iteration times are reached. If the ending condition is satisfied, the optimal solution is output. If the ending condition is not satisfied, the iteration continues, and a new population is generated through selection, intersection, and mutation, and the optimal solution is finally obtained.

According to the above process and Equation (4), the optimized model is:

(5) $$M\left(v_{c},f,a_{p}\right)=0.3Ra\left(v_{c},f,a_{p}\right)-0.7\eta_{p}\left(v_{c},f,a_{p}\right)$$

And the constraint equation is:

(6) $$\left\{\begin{array}{c} 100\;m/\min\leq v_{c}\leq 200\;m/\min\\ 0.05\;mm/r\leq f\leq 0.15\;mm/r\\ 0.1\;mm\leq a_{p}\leq 0.2\;mm\\ \begin{array}{l} 0\leq \eta_{p}\leq 67.23\\ 0\leq Ra\leq 0.8 \end{array} \end{array}\right.$$

After several iterations, the fitness function tends to be gentle and reaches the convergence state, and the optimal solution can be obtained as follows: *v_c_* = 126 m/min, *f* = 0.11 mm/r, *a_p_* = 0.14 mm. Meanwhile, the surface roughness and remnant depth ratio reach the optimal state, which is 0.489 μm and 64.13%, respectively. From Table 2, the minimum value obtained by the experiment is 33.9%, which is smaller than that of the optimal solution. And the remnant depth ratio of the optimal solution is close to the maximum value of the experimental result.

## 4. Conclusions

In this study, RSM designed experiments to test the high-speed turning of NiTi SMA. The genetic algorithm was used to obtain the best machining parameters with multiple objectives. Based on the results, the following conclusions can be drawn:

1. The prediction models of both surface roughness and the remnant depth ratio are built, and the linear, quadratic, and cross-product terms all have a significant influence on the response variables.
2. ANOVA reveals that the impact of the feed on the surface roughness is most apparent, followed by the cutting speed and the depth of the cut. The remnant depth ratio also takes into account the depth of the cut, feed, and the cutting speed.
3. Based on the genetic algorithm, taking the minimum surface roughness and the maximum remnant depth ratio as the objectives, the multi-objective optimized solution can be obtained as follows: *v_c_* = 126 m/min, *f* = 0.11 mm/r, *a_p_* = 0.14 mm and the *R_a_* and remnant depth ratio reach was 0.489 μm and 64.13%, respectively.

## Figures and Tables

**Table 1:** The factors and levels of RSM based on BBD.

Factor	Parameter	Unit	Level		
			−1	0	1
A	Cutting speed	m/min	105	144	200
B	Feed	mm/rev	0.05	0.1	0.15
C	Depth of cut	mm	0.1	0.15	0.2

**Table 2:** Box–Behnken design and results.

No.	Cutting Parameter			*R_a_* (μm)	$\eta_{p}$ (%)
	Cutting Speed (m/min)	Feed (mm/rev)	Depth of Cut (mm)		
1	144	0.05	0.1	0.498	53.87
2	105	0.1	0.2	0.532	64.18
3	144	0.1	0.15	0.476	61.25
4	144	0.1	0.15	0.466	61.24
5	200	0.05	0.15	0.368	56.22
6	105	0.15	0.15	0.610	58.48
7	144	0.15	0.2	0.645	58.16
8	105	0.05	0.15	0.323	60.15
9	200	0.15	0.15	0.662	60.73
10	105	0.1	0.1	0.473	58.49
11	200	0.1	0.1	0.629	58.61
12	144	0.15	0.1	0.736	57.05
13	200	0.1	0.2	0.425	59.27
14	144	0.05	0.2	0.327	58.48
15	144	0.1	0.15	0.462	60.96

**Table 3:** The ANOVA and coded coefficients for the prediction model of the surface roughness.

Source	F Value	*p* Value	Remarks	Coefficient
Model	55.42	<0.0001	Significant	
A	6.11	0.056		0.0182
B	370.42	<0.0001	Significant	0.1421
C	47.46	0.001	Significant	−0.0508
A^2^	0.41	0.548		−0.007
B^2^	7.49	0.041	Significant	0.029
C^2^	24.45	0.004	Significant	0.053
AB	0.03	0.873		0.001
AC	39.64	0.001	Significant	−0.065
BC	3.67	0.144		0.020

**Table 4:** The ANOVA and coded coefficients for the prediction model of the remnant depth ratio.

Source	F Value	*p* Value	Remarks	Coefficient
Model	35.14	0.001	Significant	
A	6.11	0.007	Significant	−0.809
B	370.42	0.011	Significant	0.712
C	47.46	<0.0001	Significant	1.509
A^2^	0.41	0.122		0.496
B^2^	7.49	<0.0001	Significant	−2.751
C^2^	24.45	0.002	Significant	−1.509
AB	0.03	0.002	Significant	1.545
AC	39.64	0.004	Significant	−1.258
BC	3.67	0.019	Significant	−0.875

## Data Availability

Data is available upon request from the corresponding author. There was no obligation to make the data publicly available during the course of this project.
